# Testing of behavioral and cognitive development in rats after prenatal exposure to 1800 and 2400 MHz radiofrequency fields

**DOI:** 10.1093/jrr/rrz097

**Published:** 2020-01-11

**Authors:** Zhi-qiang Li, Yuan Zhang, Yue-Meng Wan, Qiong Zhou, Chang Liu, Hui-Xin Wu, Yun-Zheng Mu, Yue-Feng He, Ritika Rauniyar, Xi-Nan Wu

**Affiliations:** 1 The School of Public Health, Kunming Medical University, Kunming, Yunnan, 650500, China; 2 The Biomedical engineering research center, Kunming Medical University, Kunming Yunnan, 650500, China; 3 International Education School, Kunming Medical University, Kunming Yunnan, 650500, China

**Keywords:** radiofrequency field, physiological and cognitive development, hippocampus, NMDARs

## Abstract

The objective of the study was to explore the effects of behavioral and cognitive development in rats after prenatal exposure to 1800 and 2400 MHz radiofrequency fields. Pregnant female rats were exposed to radiofrequency fields beginning on the 21st day of pregnancy. The indicators of physiological and behavioral development were observed and measured in the offspring rats: Y maze measured at 3-weeks postnatal, open field at 7-weeks postnatal, and the expression of *N*-methyl-D-aspartate receptors (NMDARs) measured by reverse transcription-PCR in the hippocampus at 9-weeks postnatal. The body weight of the 1800 MHz group and the 1800 MHz + WiFi group showed a downward trend. The eye opening time of newborn rats was much earlier in the WiFi group than in the control group. Compared to the control group, the overall path length of the 1800 MHz + WiFi group was shortened and the stationary time was delayed. The path length of the WiFi group was shortened and the average velocity was increased in the error arm. The 1800 MHz + WiFi group displayed an increased trend in path length, duration, entry times and stationary time in the central area. In both the 1800 MHz + WiFi and WiFi groups, NR2A and NR2B expression was down-regulated, while NR2D, NR3A and NR3B were up-regulated. Moreover, NR1 and NR2C in the WiFi group were also up-regulated. Prenatal exposure to 1800 MHz and WiFi radiofrequency may affect the behavioral and cognitive development of offspring rats, which may be associated with altered mRNA expression of NMDARs in the hippocampus.

## INTRODUCTION

Electromagnetic fields (EMF) of all frequencies are among the fastest growing public nuisances. With the advancement of technology, all populations are now exposed to varying degrees of EMF and electromagnetic radiation levels continue to rise. The speculation and anxiety about electromagnetic radiation is spreading among people [1]. Until December 2018, there were about 1.57 billion telephone users in China, up to 112.2 mobile phones per 100 persons [2]. Mobile phones are low-powered radiofrequency (RF) transmitters. The main wavebands adopted in mobile phones and Wi-Fi networks are in the high frequency (HF) range of the electromagnetic spectrum (several GHz). The frequency range of HF is from 100 KHz to 300 GHz [3], which is included in the frequency range of the RF (in the range between 100 kHz to 300 GHz) [4]. The HF range enables mobile phones to call and transfer data, including communication through the internet. The specific frequency band used differs among countries and technologies (GSM, UMTS, 4G, 5G etc.) [5]. In this study, GSM 1800 MHz is selected as the mobile phone signal frequency, while the Wi-Fi signal frequency is 2400 MHz, both within the HF range.

The EMF produced by mobile phones is classified by the International Agency for Research on Cancer (IARC) as possibly carcinogenic to humans (Group 2B). A large number of studies have shown that exposure to EMF may be associated with damage to the nervous, reproductive, immune and visual systems of animals and humans [6–13]. Pregnant females and children are more sensitive populations. Possible RF effects on children were specifically raised by the UK’s independent expert group on mobile telephones [14]. The central nervous system (CNS) was considered potentially one of the most susceptible tissues and organs that continue to develop during childhood. The hippocampus is an important part of the mammalian CNS and the structural basis of learning and memory function [15–16]. The hippocampus is closely related to the abilities of learning memory and spatial localization and is also one of the target tissues of RF radiation [17]. According to the literature reports, exposure to a certain dose of EMF during the pregnancy may have an impact on the ability of the offspring to learn and remember [18–19]. But these results are inconsistent, which is also significant for our research.

In the present study, we aimed to investigate the effects of prolonged exposure of pregnant rats to HF on the development and maturation of the cognitive function of their offspring, using physiological development indicators, behavioral tests (i.e. Y-maze or open field test) and molecular tests (i.e. cognition-related genes).

## MATERIALS AND METHODS 

### Animal experimentation

Six-week-old female Wistar rats [Shanghai Institutes for Biological Sciences, CAS, SCXK(Hu) 2017–0005] were maintained in a temperature-controlled room at 22.0°C with 60.0% humidity. After an acclimatization period of 2 weeks, the 8-week-old male and female rats were mated at 18:00 every night, and vaginal smear examination was conducted in the morning of the next day. The rats were recognized as pregnant as long as sperm were observed in vaginal smears under the microscope. Then pregnant rats were randomly divided into control group (*n* = 9) and experimental group (*n* = 9), which were further divided into the following subgroups: 1800 MHz exposure group (E1800 group; *n* = 3), 1800 MHz + WiFi exposure group (E1800 + W group; *n* = 3) and WiFi exposure group (W group; *n* = 3). All pregnant rats were exposed during 21 days of pregnancy. The exposure time was from day 0 to day 20 during pregnancy, from 20:00 every night to 8:00 in the next morning. Rats in each group had free access to drink and food during the exposure, and the environmental settings were stable. The RF field exposure status and offspring number of each group are shown in Table 1. [20]. Some growth and development indices (including body weight, body length, tail length, ear opening time, eye opening time, baby teeth appearing time) were measured in offspring rats after natural births. When the offspring rats were 3-weeks-old and 7-weeks-old, the ‘Y’ maze test and open field test were performed to observe the behavior changes. Subsequently, reverse transcription-PCR (RT-PCR) was used to detect the mRNA expression of *N*-methyl-D-aspartate receptors (NMDARs) in the hippocampus. The health condition of all rats was monitored throughout the experiment. The animal experiments were conducted in compliance with guidelines concerning the use of laboratory animals of the Kunming Medical University Animal Experimental Ethical Committee (approval number: KMMU2019043).

**Table 1:** The radiofrequency fields exposure status and offspring number of each group of pregnant rats

Group	Frequency band^a^	Power density	Number of pregnant females	Number of actual pregnancies	Number of offspring	Number randomly selected^b^	Exposure time
E1800 group	1800 MHz	1.0 mW/cm^2^	3	3	18	12	
W group	WiFi 2.4GHz	0.1 mW/cm^2^	3	2	17	12	20:00~8:00
E1800 + W group	1800 MHz + WiFi 2.4GHz	1.0 mW/cm^2^	3	3	21	12	12 h/day, 21 days
Control group	background value	30~50 μW/cm^2^	9	7	63	36	

^a^ICNIRP [18] recommended reference value of radiation protection: the limit value for public exposure to 1800 MHz is 0.9 mW/cm^2^, and the limit value for public exposure to 2.4GHz is 1.0 mW/cm^2^.

^b^Number drawn randomly by computer male:female = 1:1.

### Exposure devices

**Fig. 1.**
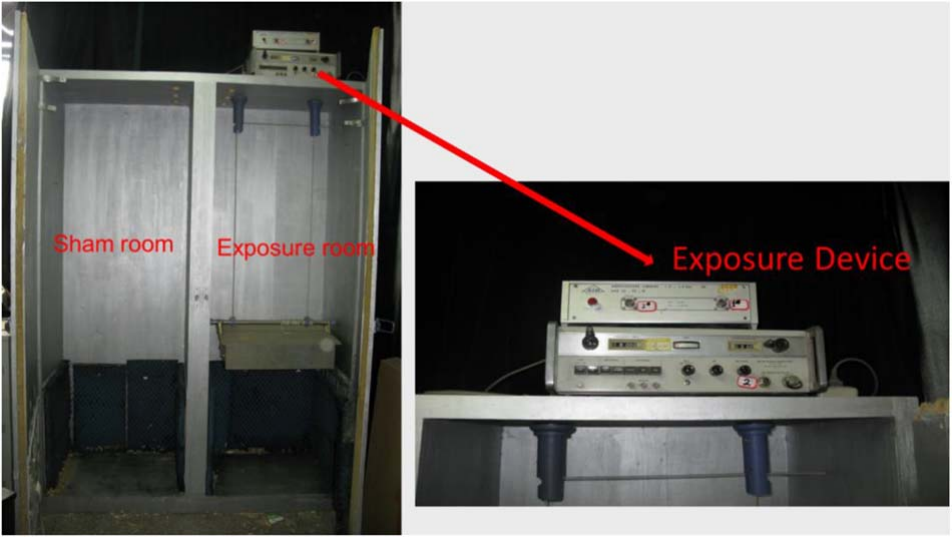
Exposure devices (length x width x height = 70 x 55 x 200 cm).

According to the standards of European digital GSM Mobile Communication provided by the German National Center for Environmental and Health Research (GSF), exposure devices are made up of a 8614A Signal Generator (0.8–2.4 GHz, Hewlett Parkard, USA) and an SCD Amplificateur Lineaire (1.3–2.6 GHz, France). Such a device was assembled by our team. Signal Generator and SCD Amplificateur Lineaire were at the top of the exposure room, and the SCD amplifier could be adjusted up and down (Fig. 1). A Narda Path Alignment System (Microwave Unit, Model 7620, USA) and a Spectrum Analyze (Hewlett Parkard 8592C, USA) were used to measure the parameters of RF fields. This measured value was the final electromagnetic field intensity at the exposure position of the pregnant rats (the detector was placed under the cage-top and drink bottle). The exposure devices were opened for 30 min before each exposure in order to maintain the stability of exposure intensity. Then, the strength of the EMFs were measured at the beginning and end of each exposure. The temperature of the exposure room was controlled at 18.0–24.0°C.

### Y maze test

**Fig. 2.**
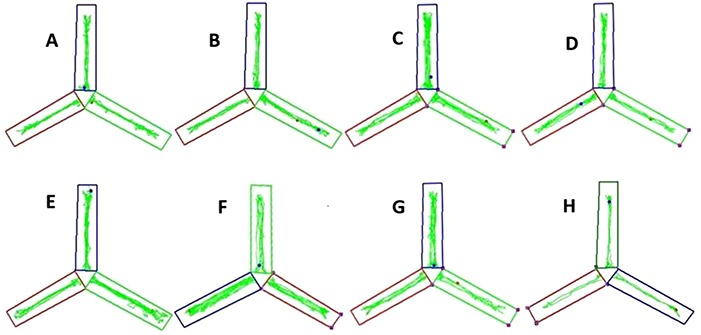
Y maze representative trajectories of offspring in each group. (**A**) and (**B**) Trajectories of 1800 MHz experimental group; (**C**) and (**D**) trajectories of 1800 MHz + WiFi experimental group; (**E**) and (**F**) trajectories of WiFi experimental group; (**G**) and (**H**) trajectories of control group.

The Y maze is made of medical black organic board with three arms: start arm, error arm and food arm. The arms are placed at 120^°^ angles to one another. The size of each arm is 60 x 15 x 15 cm (length x width x height). Y maze’s three arms were randomly named as starting arm, food arm and error arm. Rats entered through the start arm and moved freely throughout the maze (the adaptation period). Error arm was blocked by a baffle in the first stage of the experiment (the training period), and was opened in the second stage (the test period), whereas starting arm and food arm were open throughout the experiment. Lastly, after each training or test, the inner wall and bottom of the maze was wiped with medical alcohol to keep the maze clean and to prevent interference of residual odor of animals. A camera was installed 1.5 m above the maze. The whole process was recorded and analyzed by computer automatically (Fig. 2). The Y maze test was divided into three stages: adaptation period (1 day), training period (2 days) and test period (1 day). The animals were deprived of food in order to control their body weight down to 80–85% of the normal weight before the experiment.

1) Food and time setting

During the adaptation period, no food was given, and all arms were in contact with each other. The acclimation time was 8:30–10:00 , 12:30–14:00 and 18:00–20:00, each time for 10 min, for a total of 1 day. During the training period, food was given and the error arm was closed. The training time was 8:30–10:00, 12:30–14:00 and 18:00–20:00, each time for 10 min, for a total of 2 days. During the test period, the spontaneous behaviour records were analyzed. The test time was between 18:00 and 21:00.

2) Spontaneous behaviour records.

The animals were put in the end of the starting arm and the software automatically recorded and analyzed within 5 min. Indicators included the path length, duration, stationary time, average velocity, entry times and incubation time of the animals entering each arm.

### Open field test

Open field test, also known as open box test, is a method to evaluate the autonomous behavior, exploratory behavior and tension of experimental animals in new and different settings. Indicators of the autonomous behaviors and exploratory behaviors of experimental animals in an unfamiliar environment include: the occurrence, frequency and duration of some behaviors and the speed in different cognitive zones of the novel and open environment. For example, the number of urination and standing times reflect the animals’ tension. The size of the box was: 100 x 100 x 60 cm (length x width x height).

**Fig. 3.**
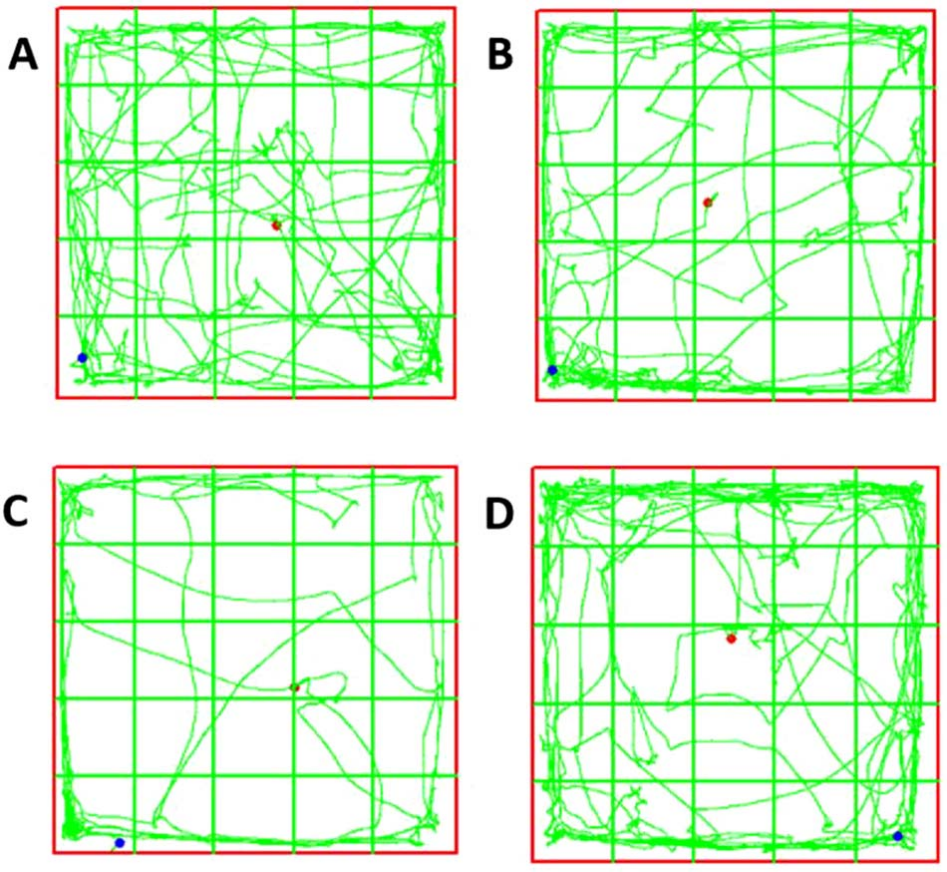
Open field representative trajectories of offspring in each group. (**A**) The trajectory of 1800 MHz experimental group; (**B**) the trajectory of the WiFi experimental group; (**C**) the trajectory of the 1800 MHz + WiFi experimental group; (**D**) the trajectory of the control group.

The computer software was used to draw virtual 25 (5 rows × 5 columns) place grids on the corresponding pictures during operation. A small grid in the center was named as the central area, the side area being 16 small grids in the periphery zone of the box’s bottom (Fig. 3). After each training or test, the inside and bottom of the box were wiped with medical alcohol to prevent the interference of residual odor. A camera was installed 1.5 m above the box and the whole process was recorded.

### RT-PCR of NMDARs in the hippocampus

Triazol reagent kit (Tiangen Company, China) was used to extract the total RNA of hippocampuses, and RT was performed using the Quant Script RT kit (Roche Company, Switzerland). PCR was performed using ABI7900HT (ABI Company, USA) according to established methods, 2^−△△CT^ values were used to indicate the relative expression of seven RNAs (NR1, NR2A−2D, NR3A−3B). Seven PCR primers were selected and prepared to analyze the changes in the hippocampuses of offspring rats (Table 2). Each sample was measured in triplicate and β-action was used as an internal control. The PCR reaction system is displayed in Table 3. The PCR conditions were: thermal denaturation 95°C × 10 s, annealing: 60°C × 10 s and elongation: 72°C × 10 s, for 45 cycles.

**Table 2:** Table showing the primers used for RT-PCR assays

Gene	Primer (5′-3′)
NMDAR1	-CAGGAGTGGAACGGAATCAT-
	-ACTTGAAGGGCTTGGAGAAC-
NMDAR2A	-AGCCATTGCTGTCTTCGTTT-
	-ATCTTGCTGGTTGTGCCTTT-
NMDAR2B	-GGCCTTCTTTGCTGTCATTTT-
	-AGTTCTTCCATCTCCCCATCTC-
NMDAR2C	-CACACCCACATGGTCAAGTTC-
	-ATGGTGACCAGCTTGCAGC-
NMDAR2D	-CGAGGATGGCTTTCTGGTGA-
	-ATACTTGAGGCGGAGGGTCTG-
NMDAR3A	-GCGGGATGCCCTACTGTT-
	-CATTTCGCCCTGGCTCTG-
NMDAR3B	-CGAGGATGGCTTTCTGGTGA-
	-ATACTTGAGGCGGAGGGTCTG-
β-Action	-TGACGTGGACATCCGCAAAG-
	-CTGGAAGGTGGACAGCGAGG-

**Table 3:** RT-PCR reaction system

Reagent	Volume(μL) 96-well plate
SYBR Green master mix	7
PCR primer mix	0.5
Diluted cDNA template	1.5
RNase-free water	6
Total volume	15

### Statistical analysis

All data analysts were blind to animal experimental group members. Continuous variables were expressed as $\overline{x}$ ±s, categorical data were expressed as M(means median). Statistical analysis was done using SPSS 17.0 statistical analysis software (SPSS). Normal distribution was achieved by log transformation for some skewed data. Comparison among the four groups was done using ANOVA or Rank Sum Test; multiple comparison was executed by Least Significant Difference - t test(LSD-t) or Student - Newman - Keuls(SNK).

## RESULTS

### Physiological development

The body weights and tail length of the 1800 MHz exposure group and the 1800 MHz + WiFi exposure group were smaller than the control group (*P* < 0.05). The eye opening time of the 1800 MHz + WiF exposure group was earlier than the WiFi exposure group (*P* < 0.05). Other physiological development indices were not different among the groups (Table 4).

**Table 4:** Physiological development indicators of each group of rats after birth ($\overline{x}$±s)

Group	Body weight(g)	Body length(cm)	Tail length(cm)	Ear opening time (day)	Grow new teeth time(day)	Eye opening time (day)
E1800 group	5.91 ± 0.40^a^	4.95 ± 0.16	1.51 ± 0.06^a^	4.17 ± 0.39	8.33 ± 1.07	16.17 ± 0.39
W group	6.17 ± 0.22	4.91 ± 0.14	1.55 ± 0.10	4.00 ± 0.00	7.50 ± 0.52	17.08 ± 1.24
E1800 + W group	5.50 ± 1.13^a,b^	5.15 ± 0.76	1.48 ± 0.11^a^	4.08 ± 0.29	8.25 ± 0.45	15.92 ± 0.79^b^
Control Group	6.41 ± 0.47	5.06 ± 0.19	1.60 ± 0.09	4.08 ± 0.28	8.47 ± 1.32	16.50 ± 0.66
F/H	7.634	1.261	6.174	2.152 (H)	7.055 (H)	11.252 (H)
*P*	0.000	0.295	0.001	0.542	0.070	0.010

^a^Significant difference (*P* < 0.05) when compared with the control group.

^b^Significant difference (*P* < 0.05) when compared with the E1800 + W group.

### Behavioral changes

*Y Maze test (* *Table 5* ***)***

**Table 5:** The experimental results of the Y maze test in 3-week-old offspring rats ($\overline{x}$±s)

		E1800 group	W group	E1800 + W group	Control Group	F	*P*
		(*n* = 12)	(*n* = 12)	(*n* = 12)	(*n* = 36)		
Overall	Path length (mm)	41242.23 ± 3603.66^a^	36334.30 ± 4026.66	32868.81 ± 7249.57^a,b^	39397.54 ± 8797.91^b^	3.423	0.022
	average velocity (mm/s)	137.80 ± 11.92	133.98 ± 15.98	119.76 ± 25.25	143.22 ± 33.51	2.269	0.088
	stationary time (s)	52.18 ± 8.15^a^	54.86 ± 9.76^b^	69.60 ± 19.38^a,b^	47.99 ± 14.97	6.999	0.000
	path length (mm)	13362.73 ± 1753.68^a^	11946.30 ± 1878.78	10168.11 ± 2720.62^a,b^	13318.87 ± 4308.09^b^	2.866	0.043
	duration (s)	103.08 ± 20.66	83.50 ± 18.68	82.83 ± 22.51	92.29 ± 24.99	2.070	0.112
Starting arm	Average velocity (mm/s)	131.76 ± 16.08	146.74 ± 23.28	127.41 ± 37.10	147.99 ± 44.94	1.285	0.287
	Entry times	6.75 ± 1.96	7.50 ± 1.57	6.00 ± 2.17	7.61 ± 2.71	1.611	0.195
	Incubation time (s)	5.40 ± 5.85	2.41 ± 5.97	18.18 ± 34.54	6.32 ± 16.71	1.709	0.173
	Stationary time (s)	19.36 ± 4.84	14.63 ± 6.51	21.23 ± 12.67	16.86 ± 7.46	1.638	0.189
	Path length (mm)	12328.63 ± 2165.16	10235.59 ± 1736.80	10512.81 ± 3184.59	11921.48 ± 3167.36	1.882	0.141
	Duration (s)	82.90 ± 16.50^a^	60.64 ± 10.44^a,b,c^	86.81 ± 33.84^b^	76.38 ± 23.90^c^	2.951	0.039
Error arm	Average velocity (mm/s)	150.04 ± 14.87^a^	171.23 ± 31.24^a,b^	127.14 ± 30.69^b^	165.42 ± 45.40	3.850	0.013
	Entry times	7.33 ± 1.72^a^	7.50 ± 1.68^b^	5.75 ± 1.66^a,b,c^	7.53 ± 2.09^c^	2.790	0.047
	Incubation time (s)	3.92 ± 2.93	7.857 ± 5.86	6.60 ± 11.23	4.83 ± 4.56	1.114	0.349
	Stationary time (s)	12.70 ± 4.19^a^	9.00 ± 4.88^b^	21.99 ± 14.01^a,b,c^	11.62 ± 7.86^c^	5.844	0.001
	Path length (mm)	15545.92 ± 1397.12	141527.50 ± 2605.66	12187.95 ± 3469.88	14154.57 ± 3160.19	2.704	0.052
	Duration (s)	113.24 ± 19.22	128.08 ± 30.44	104.35 ± 33.36	107.79 ± 29.58	1.763	0.162
Food arm	Average velocity (mm/s)	140.87 ± 27.93	113.52 ± 21.03	121.80 ± 31.22	137.26 ± 35.22	2.434	0.072
	Entry times	8.08 ± 1.38	8.25 ± 2.01	6.67 ± 1.88	8.03 ± 2.70	1.296	0.283
	Incubation time (s)	1.47 ± 1.69	3.30 ± 3.60	2.73 ± 2.44	3.77 ± 3.83	1.514	0.219
	Stationary time (s)	20.11 ± 7.70^a^	31.24 ± 11.71^a,b^	26.39 ± 11.85	19.52 ± 12.00^b^	3.848	0.013

^a,b,c^Significant difference (*P* < 0.05) when comparing the groups with each other.

Compared to control groups, the overall path length of the 1800 MHz + WiFi exposure group was shortened, and the stationary time was increased. The number of entry times decreased and the stationary time increased in the error arm (*P* < 0.05). Compared to control groups，the path length of the WiFi exposure group was shortened and the average velocity was increased in the error arm (*P* < 0.05). The stationary time was increased among the WiFi exposure group in the food arm (*P* < 0.05).

*Open field test (* *Table 6* ***)***

**Table 6:** Results of open field test in 7-week-old offspring rats ($\overline{x}$±s)

		E1800 group	W group	E1800 + W group	Control Group	F/H	*P*
		(*n* = 12)	(*n* = 12)	(*n* = 12)	(*n* = 36)		
	Path length (mm)	74099.75 ± 21184.41	69117.52 ± 20325.16	55240.38 ± 9283.19	77339.77 ± 37461.84	1.730	0.169
Overall	Average velocity (mm/s)	124.11 ± 35.64	115.36 ± 33.89	92.08 ± 15.48	128.93 ± 62.43	1.735	0.168
	Stationary time (s)	197.95 ± 60.26^a^	208.74 ± 65.77^b^	264.75 ± 43.07^a,b,c^	192.40 ± 57.75^c^	4.906	0.004
	Path length (mm)	733.51 ± 491.48^a^	991.58 ± 792.44^b^	20707.12 ± 25554.94^a,b,c^	931.27 ± 757.27^c^	12.325	0.000
	Duration (s)	2.15 ± 2.06^a^	3.31 ± 3.79^b^	242.62 ± 299.36^a,b,c^	3.90 ± 5.50^c^	13.139	0.000
Central zone	Average velocity (mm/s)	446.81 ± 279.60	398.30 ± 231.59	385.44 ± 466.37	332.57 ± 245.06	0.509	0.677
	Entry time (s)	2.83 ± 2.13^a^	3.58 ± 3.12^b^	7.00 ± 7.53*^b,c^	3.44 ± 2.76^c^	2.973	0.038
	Incubation time (s)	163.00 ± 196.85	52.07 ± 55.20	129.47 ± 223.55	171.66 ± 176.59	1.465	0.232
	Stationary time (s)	0.33 ± 0.69^a^	0.21 ± 0.46^b^	106.29 ± 135.62^a,b,c^	0.67 ± 2.57^c^	12.520	0.000
	Path length (mm)	63016.38 ± 15613.00^a^	60134.34 ± 16014.54^b^	29807.02 ± 26213.97^a,b,c^	66514.84 ± 30681.35^c^	6.141	0.001
	Duration (s)	552.57 ± 35.95^a^	562.82 ± 16.59^b^	333.97 ± 293.21^a,b,c^	550.10 ± 28.53^c^	11.015	0.000
Periphery zone	Average velocity (mm/s)	115.41 ± 33.69	107.51 ± 30.58	163.41 ± 168.26	121.61 ± 57.49	1.153	0.334
	Entry time (s)	30.33 ± 12.87^a^	24.67 ± 11.20^b^	12.50 ± 9.55^a,b,c^	27.39 ± 13.72^c^	5.087	0.003
	Incubation Time (s)	0.05 ± 0.18^a^	0.31 ± 0.50^b^	68.09 ± 170.60^a,b,c^	0.18 ± 0.27^c^	3.265	0.027
	Stationary time (s)	187.76 ± 67.27	202.01 ± 68.20	150.72 ± 137.65	180.26 ± 59.37	0.881	0.455
Events	Number of grooming	19.50	42.33	39.08	39.36^d^	9.874 (H)	0.020
	Number of standing	29.33	29.88	35.54	41.75	4.986 (H)	0.173
	Number of micturition	36.92	31.00	36.92	38.06	2.664 (H)	0.446
	Number of stool	41.17	33.33	38.00	35.54	1.695 (H)	0.638

^a,b,c^Significant difference (*P* < 0.05) when compared with the two groups each other; ^d^significant difference (*P* < 0.05) when compared with the other three groups in number of grooming.

Compared to other groups, the 1800 MHz + WiFi exposure group showed an increased trend in the path length, duration, entry times and stationary time in the central zone. In the periphery zone, the path length, duration and entry times of the offspring rats of the 1800 MHz + WiFi exposure group were lower than those in other groups (*P* < 0.05), and the incubation time was longer than other groups (*P* < 0.05). There was no difference among other groups.

### mRNA levels of seven subunits of the NMDAR gene in the hippocampus of offspring rats (Fig. 4)

In the 1800 MHz + WiFi exposure group, the RNA relative expression levels of NR2A and NR2B were lower than in other groups (*P* < 0.05), and RNA relative expression of NR2D, NR3A and NR3B were higher than in the control group (*P* < 0.05). In the WiFi exposure group, RNA relative expression of NR2A and NR2B were lower than in the control group (*P* < 0.05), and RNA relative expression of NR1, NR2C, NR2D, NR3A and NR3B were higher than in the control group (*P* < 0.05).

## DISCUSSION

The embryonic development stage is sensitive to the outside environment and susceptible to external harmful factors. Body weight, tail length, eye opening time etc. are all important and commonly used indicators for developmental toxicity, which was also measured in this study. The results showed that the body weights of the 1800 MHz exposure group and the 1800 MHz + WiFi exposure group were lower than that of the control group, and the body weight of the 1800 MHz + WiFi exposure group was lower than that of the WiFi exposure group. However, the teething time of the 1800 MHz + WiFi exposed group was earlier than that of the WiFi exposed group. These results suggest that there may be differences in the influence of mobile phone and WiFi RF exposure during pregnancy on the growth and development of offspring, but the results need to be further verified.

The Y maze experiment is a common experiment used to study animal behavior, and is widely used in the neurobiology field of spatial learning and memory, including addictive substance dependence, mental disease, drug experiment, etc. [21–23]. In the Y maze experiment the weight of animals is controlled by fasting before the experiment, forcing them to take the initiative to look for food in the maze during the training phase, and make them learn and memorize the route by constantly strengthening the search route. It mainly reflects the ability of animals to get spatial and food odor information, integrate memory, etc., and the brain regions involved include mainly the medial frontal cortex, hippocampus etc. [24]. The activity intensity of offspring rats in the maze (movement distance, speed, resting time, etc.) also indicated their ‘curiosity’ about their environment. The results of Y maze testing of 3-week-old offspring rats showed that the total path length of activity was shortened and the total stationary time was increased in the 1800 MHz + WiFi exposure group as compared to the control group. The entry frequency of the error arm decreases and the stationary time increases. The results showed that the memory accuracy and exploratory ability of the offspring in this group were poor. It is suggested that exposure to mobile phone and WiFi during pregnancy may have negative effects on spatial learning and memory ability of infant offspring. In the WiFi exposed group, the duration was shortened and the average velocity was increased in the error arm. The stationary time increased in the feeding arm. The accuracy of memory was relatively higher than in the control group. This may suggests that the exposure to WiFi RF during pregnancy has a positive effect on the spatial learning and memory ability of the offspring.

Open field experiments are mainly used to evaluate the autonomous behavior, exploratory behavior and tension of experimental animals in new environments. Some indicators in the experimental animals were used to reflect the autonomous behaviors and exploratory behaviors in unfamiliar environments. For example, the frequency of urination and standing was used to reflect the sense of tension in offspring rats. The specific parameters measured in this open field experiment include: distance, speed and time in the central area and the peripheral area, the number of standing urinations, the number of stool particles, and other behaviors, including scratching the head, licking the feet and grooming, which indicate the spontaneous behavior of the animal. The open field experiment results of 7-week-old offspring rats showed that the path length, duration, entry times and stationary time in the central area for the 1800 MHz + WiFi exposed group were all higher than those of other groups, whereas the distance, path length, duration and entry times in the peripheral area were lower than those in other groups. The offspring result suggests that there is a correlation between the prenatal exposure to mobile phone plus WiFi RF and the lower enthusiasm for exploration and the emotions of anxiety and fear in the new environment. With 9.417 GHz exposed mice during pregnancy, the results of Zhang *et al*. [25] are the opposite of this. They found that the activity of offspring mice decreased in the open field central area, but anxiety-related behaviors increased.

Glutamate (Glu) must bind to its receptor to function as an excitatory neurotransmitter. Ionic Glu receptors mainly include NMDAR, α-amino-3-hydroxy-5-methyl-4-isoxazole-propionc acid receptor (AMPAR) subtype and kainite receptor (KAR). These receptors have different subunits that together form the glutamate ion receptor family. They are closely related to the formation of the CNS during animal development and the plasticity of synaptic transmission efficiency, such as long-term potentiation (LTP) and long-term depression (LTD) [26–27].

Glu is the most abundant amino acid in the mammalian brain and mediates a series of high-level neural activities by binding to NMDARs. The NMDAR is a double-gated ion channel Ion channels controlled by voltage and ligand, consisting of seven known subunits (NR1, NR2A−2D, NR3A−3B), forming a tetrameric complex and interacting with intracellular cytoskeletal proteins. It is mainly located in the post synaptic density (PSD), which plays an important role in synaptic formation, maintenance and transmission plasticity to regulate learning and memory. NMDARs, when combined with Glu, can transform synaptic electrical signals that convert pre-electrical signals into Ca^2+^ signals, thus initiating a series of biochemical reactions, leading to changes in synaptic plasticity and membrane properties, leading to LTP, and thus regulating learning and memory. So, the abnormal expression of NMDAR mRNA may affect learning and memory among rats. Datwani *et al*. reported that NMDARs mediated the plasticity of cerebral cortex thalamic–cortical axons [28]. Giza *et al*. found that immature traumatic brain injury could reduce NR2A expression [29]. Some researchers knocked out the NMDAR1 gene in the mouse striatum and found that the morphology of neurons in the mouse striatum did not change significantly, but the learning ability and the LTP in dorsal striatum and the LTD in ventral striatum were interfered with [30]. It is known that the selective expression of NR1 and NR2B subunits of NMDAR, the composition of subunit heteromers, and the phosphorylation status of subunits all affect the function of NMDARs.

**Fig. 4.**
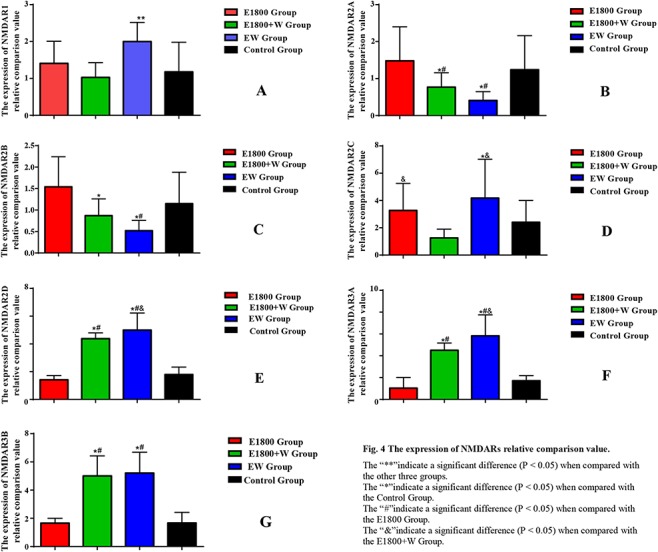
The expression of NMDARs relative comparison value. ^**^ Significant difference (*P* < 0.05) when compared with the other three groups; ^*^ significant difference (*P* < 0.05) when compared with the control group; ^#^ significant difference (*P* < 0.05) when compared with the E1800 group; ^&^ significant difference (*P* < 0.05) when compared with the E1800 + W group.

The relative expression levels of seven NMDARs subunit mRNAs in the hippocampal tissues of offspring rats showed that the mRNA relative expression of NR2A and NR2B in the 1800 MHz + WiFi exposure group was down-regulated. In the WiFi exposure group, the mRNA relative expression of NR2D, NR3A and NR3B were up-regulated, while NR2D, NR3A were down-regulated. In the E1800+W exposure group, NR1, NR2C, NR2D, NR3A and NR3B were up-regulated. These results suggest that the exposure to mobile phone signal plus WiFi RF or independent WiFi RF during pregnancy may disrupt the expression of the NMDAR gene in the offspring hippocampus.

Learning and memory are higher neurological functions in animals, and NMDA receptors in the hippocampus are necessary for memory acquisition and/or consolidation. Injecting NMDAR antagonists into the hippocampus of rodents can cause spatial memory impairment [31]. NMDAR is a hetero-tetramer composed of two essential subunits of NR1 and two regulatory subunits of NR2 (A−D) or NR3 (A−B). NR2A and NR2B are the main regulatory subunits in the CNS region involved in cognitive function, such as the hippocampus and prefrontal cortex (PFC) [32, 33]. The expression of these regulatory subunits is dynamic and strictly regulated [33, 34]. NR2B is the main regulatory subunit in embryonic development, so NR2B plays a dominant role in this period, especially in the hippocampus and PFC [35]. In the early postnatal period, NR2A transcription and translation increased, while NR2B expression remained unchanged. Therefore, the ratio of NR2A:NR2B is increased at the stage [36]. This change of NR2A:NR2B ratio is also known as the enlightening switch of NMDAR [33]. ‘Normal expression and distribution of NMDAR’ is a necessary condition for LTP induction [37].

In this experiment, the expression of NMDAR was disturbed and its structure may have changed. In the 1800 MHz + WiFi exposure group, the mRNA relative expression of NR2A and NR2B was down-regulated, but NR2D, NR3A and NR3B were up-regulated. In the 1800 MHz exposure group, the mRNA relative expression levels of NR2C was up-regulated. In the WiFi exposed group, the mRNA relative expression levels of NR2A and NR2B was down-regulated, but the mRNA relative expression of NR1, NR2C, NR2D, NR3A and NR3B was up-regulated. These results suggest that prenatal exposure to cell phone signals and/or WiFi RF may disrupt the expression of NMDARs in the offspring hippocampus. The result of Xu *et al*. [38] was similar to ours in that NR2A and NR2B were not changed through testing hippocampal neurons chronic exposure to 1800 MHz microwaves. But, Seymen *et al*. [39] found that NR2B was increased in the cerebrum and the cerebellum after exposure to GSM radiation for long periods. Another report showed acute exposure to 900 MHz microwaves activated NR2A degradation in the hippocampus [40]. Our results showed that WiFi had more effect on the expression of NMDAR than 1800 MHz, but the underlying mechanism needs to be explored in our future research.

Of course, there are many other factors that may affect learning and memory, such as the complexity of the brain’s own processing of information and the uncertainty of the biological effects of electromagnetic waves (mainly non-thermal effects). These factors may be related to the mechanism of NMDAR expression change. Because of differences between species, animal studies can often be expected to provide qualitative information regarding potential health outcomes, but the data may not be extrapolated to provide quantitative estimates of risk. So, the effects on learning and memory of human prenatal exposure to RF are still to be discovered in future research.
